# Pd on thermo-responsive composite of silica-coated carbon nanotube and 1-vinyl-3-butylimidazolium-based ionic liquid copolymers as an efficient catalyst for hydrogenation of nitro compounds

**DOI:** 10.1038/s41598-022-07708-0

**Published:** 2022-03-10

**Authors:** Samahe Sadjadi, Neda Abedian-Dehaghani, Majid M. Heravi

**Affiliations:** 1 Gas Conversion Department, Faculty of Petrochemicals, Iran Polymer and Petrochemical Institute, PO Box 14975-112, Tehran, Iran; 2 Department of Chemistry, School of Physics and Chemistry, Alzahra University, PO Box 1993891176, Vanak, Tehran, Iran

**Keywords:** Catalysis, Green chemistry, Organic chemistry

## Abstract

In this work, an ionic liquid-containing thermo-responsive heterogeneous catalyst with utility for promoting hydrogenation of nitro-compounds in aqueous media is developed. To prepare the catalyst, silica-coated carbon nanotubes were synthesized and vinyl-functionalized. The resulted compound was then polymerized with 1-viny-3-butylimidazolium bromide and *N*-isopropylacrylamide. The obtained ionic liquid-containing thermo-responsive composite was palladated via wet-impregnation method to give the final catalyst. Study of the performance of the catalyst confirmed high catalytic activity of the catalyst at temperature above the lower critical solution temperature. Furthermore, the catalyst was highly recyclable and showed negligible Pd leaching upon recycling. Broad substrate scope and selectivity of the catalyst towards reduction of nitro functionality were also confirmed. Furthermore, hot filtration test implied the heterogeneous nature of the catalysis. The comparison of the activity of Pd/CNT-P with some control catalysts approved the importance of hybridization of P and CNT and the presence of ionic liquid for the catalytic activity.

## Introduction

Smart polymers are polymeric compounds^1–3^ that respond to the external stimuli, such as mechanical force, light^4^, temperature^5,6^, pH^7^ etc. This class of polymers has gained considerable attention and has been successfully applied in diverse range of applications, including catalysis, thermochromic and electrochromic materials, biomedical fields, matrix chemistry, etc^8,9^. Poly (*N*-isopropylacrylamide), PNIPAM, is a well-known thermo-responsive polymer that in response to temperature can generate a phase-separation in the aqueous solution at the lower critical solution temperature (LCST)^10^. In fact, the hydrogen bonds between this polymer and water can result in dehydration and phase separation^10,11^.

Among various carbon nanomaterials, carbon nanotubes^12–14^, CNTs, have received significant attention. These compounds benefit from some excellent properties, such as inertness, high surface area, thermal and chemical stability, electric conductivity and tune-ability. These features attracted many scientists to utilize CNTs in various research fields, such as catalysis. One of the drawbacks of CNTs for the catalysis is their low wet-ability. This characteristic restricted the use of CNTs for the reactions in the aqueous media^15–18^. To furnish a solution to this issue, CNT can be functionalized or hybridized with hydrophilic moieties^19^.

Reduction of nitro functionality to amino group is a key chemical transformation that can be extensively utilized for the synthesis of complex chemicals and drugs^20–22^. Moreover, this reduction reaction can be exploited for de-colorization of dyes and waste water treatment. This catalytic process proceeds in the presence of conventional hydrogenation catalysts, such as Pd nanoparticles. To reduce the required content of the precious metals and achieving fine nanoparticle size and high dispersion, supporting materials, such as clays^24^ and carbohydrates^25^ are employed.

Mostly, supporting materials are modified by introduction of functional groups on their surface. This can be conducted through covalent or non-covalent approaches^26^. In this regard, one of the most investigated functional groups is ionic liquid, IL^27–29^ that is an organic salt with low toxicity^27,30,31^. As ILs are electrically charged species, they can be a promising compounds to provide electrostatic interactions with metallic nanoparticles and stabilizing them on the support. On the other hand, these compounds can also exhibit catalytic activity^32,33^. To date various IL-based catalysts have been developed for various chemical transformations^34–39^.

**Figure 1:**
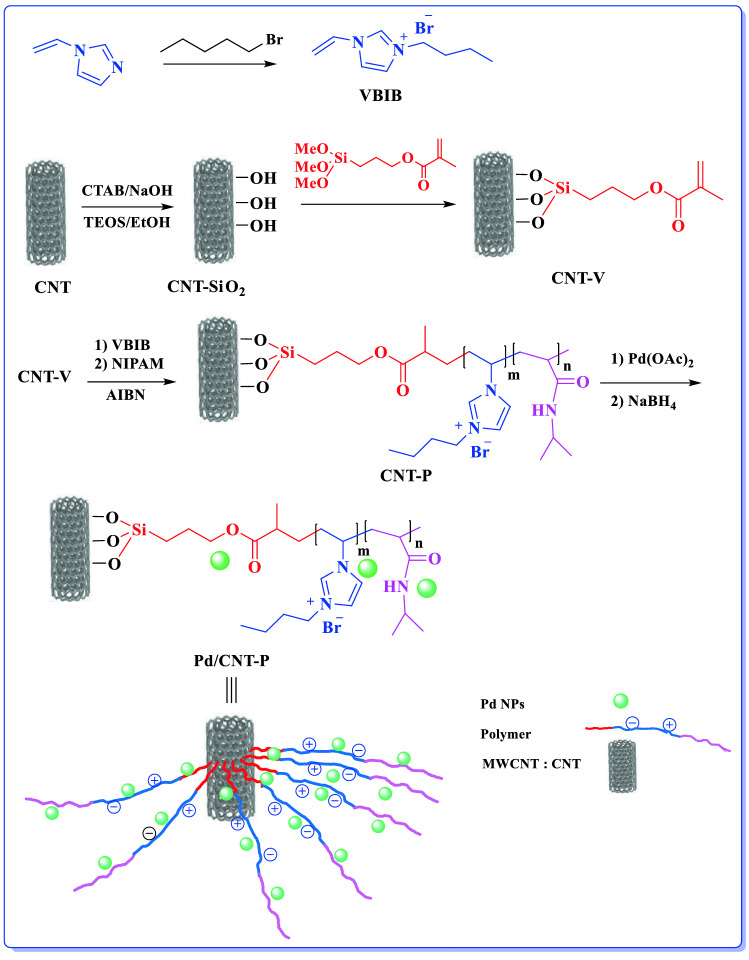
Schematic procedure for the preparation of Pd/CNT-P.

In the pursuit of our research on the heterogeneous catalysts^24,40–45^, in this project we wish to report a novel heterogeneous catalyst, Pd/CNT-P, with utility for hydrogenation of nitro compounds under mild reaction condition. To prepare the catalyst, silica-coated CNT has been synthesized and then vinyl functionalized. The resulted compound was then polymerized with the as-prepared 1-viny-3-butylimidazolium bromide (VBIB) and *N*-isopropylacrylamide (NIPAM) to furnish a thermo-responsive composite, which was subsequently palladated to give the hydrogenation catalyst (Fig. 1).

## Result and discussion

### Structure of the catalyst

**Figure 2:**
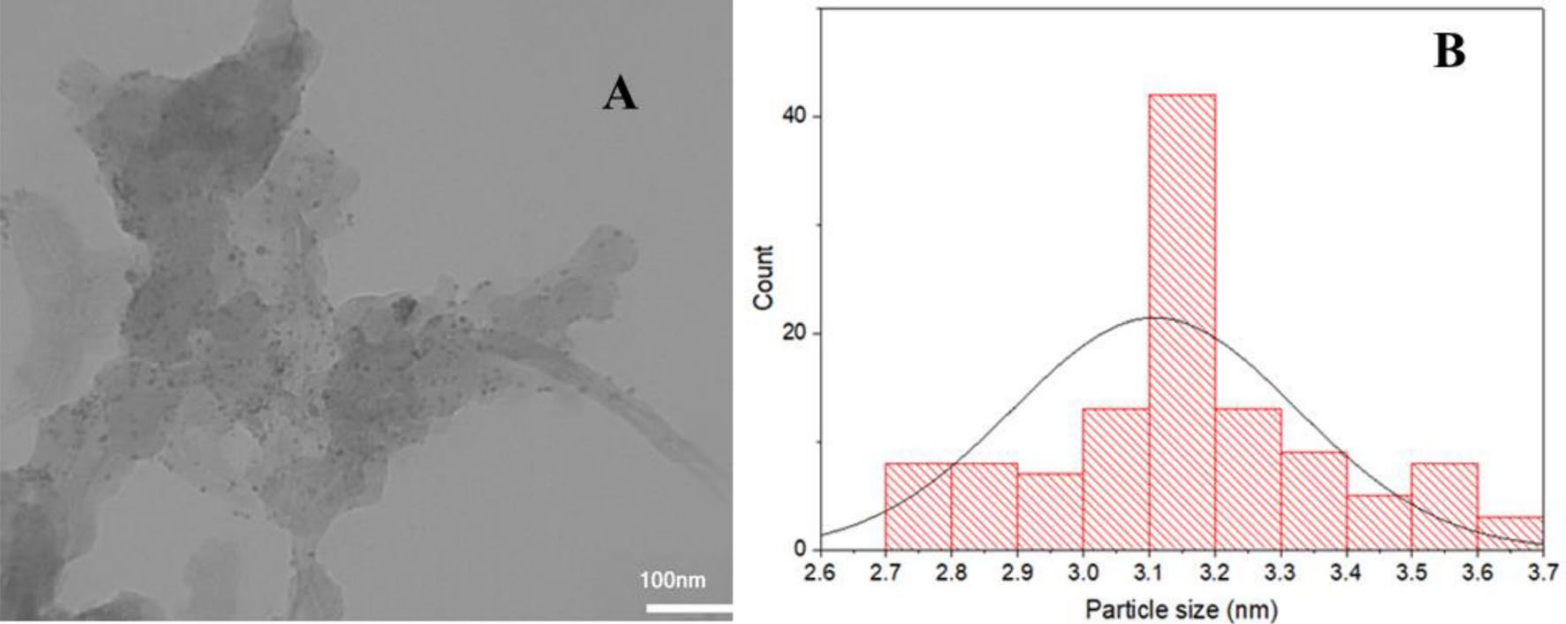
(**A**) TEM image of Pd/CNT-P and (**B**) particle size distribution of Pd nanoparticles.

To study the morphology of Pd/CNT-P, it was analyzed via TEM. In TEM image of Pd/CNT-P (Fig. 2), polymeric moiety on the CNT tubes can be discerned. On the other hand, fine Pd nanoparticles (average diameter of 4.0 ± 0.1 nm) can be seen on the CNT-P.

**Figure 3:**
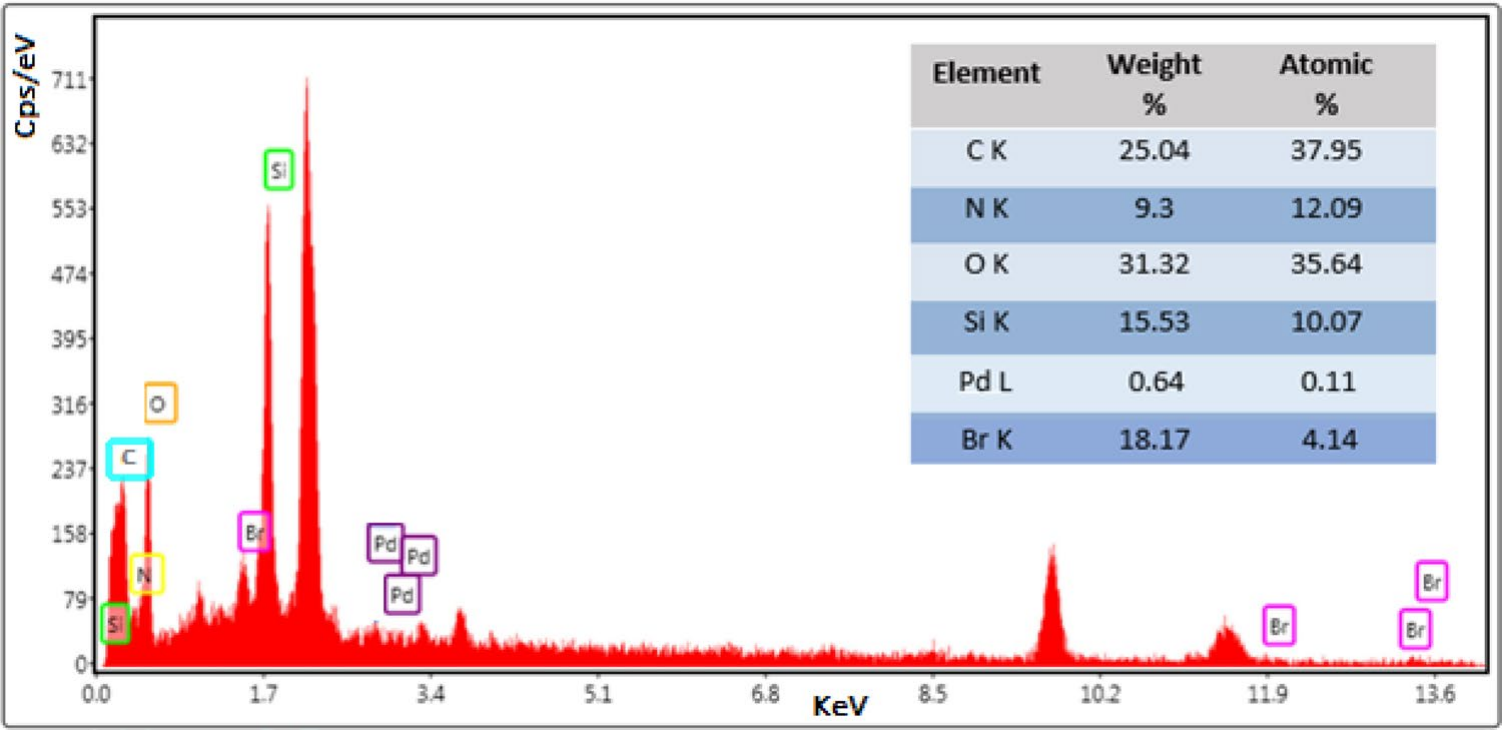
EDS analysis of Pd/CNT-P.

Pd/CNT-P was also characterized via EDS and elemental mapping analyses. As depicted in Fig. 3, the as-prepared catalyst contains C, N, O, Si, Pd and Br atoms. C, O and Si atoms can represent CNT-SiO_2_, while C, N, O and Br can be assigned to the P component. The presence of Pd also can indicate stabilization of Pd nanoparticles on CNT-P.

**Figure 4:**
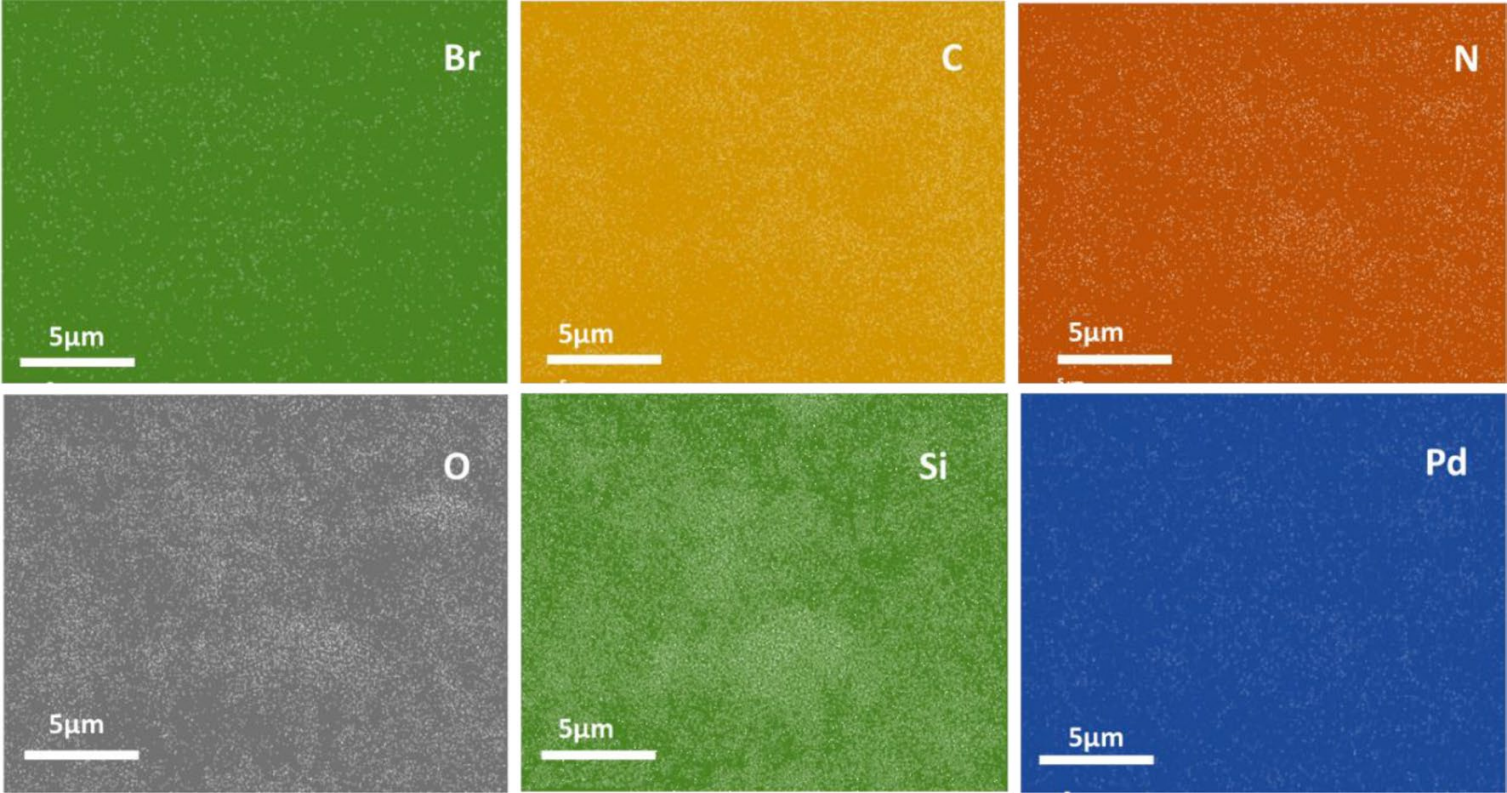
Elemental mapping of Pd/CNT-P.

Elemental mapping analysis of Pd/CNT-P, Fig. 4, showed uniform dispersion of Pd, N and Br atoms. This observation implies that both polymeric component (P) and Pd nanoparticles have been distributed of CNT-SiO_2_ homogeneously.

**Figure 5:**
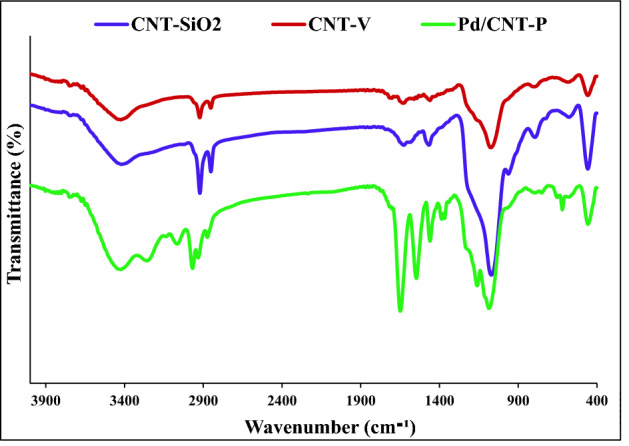
FTIR spectra of CNT-SiO_2_, CNT-V and Pd/CNT-P.

To confirm successful formation of CNT-SiO_2_, CNT-V and Pd/CNT-P, their FTIR spectrum has been recorded. As shown in Fig. 5, CNT-SiO_2_ showed the absorbance bands at 1085 cm^−1^ (Si–O-Si), 3410 cm^−1^ (–OH), 1463 cm^−1^ (–C–O), 2920 and 2850 cm^−1^ (–CH_2_ stretching). Moreover, the band at 1631 cm^−1^ can be related to the –COOH functionality of the used CNT. CNT-V FTIR spectrum also exhibited the characteristic bands of CNT-SiO_2_, affirming the stability of CNT-SiO_2_ upon functionalization. Moreover, the appearance of a small band at 1703 cm^–1^ (–C = O) can confirm conjugation of TMSPMA. In the FTIR spectrum of Pd/CNT-P, all of the characteristic absorbance bands of CNT-SiO_2_ can be detected. Moreover, the appearance of sharp bands at 1645 cm^−1^ (amidic –C=O) and 1546 cm^−1^ (–C=N) can confirm conjugation of thermo-responsive co-polymer.

**Figure 6:**
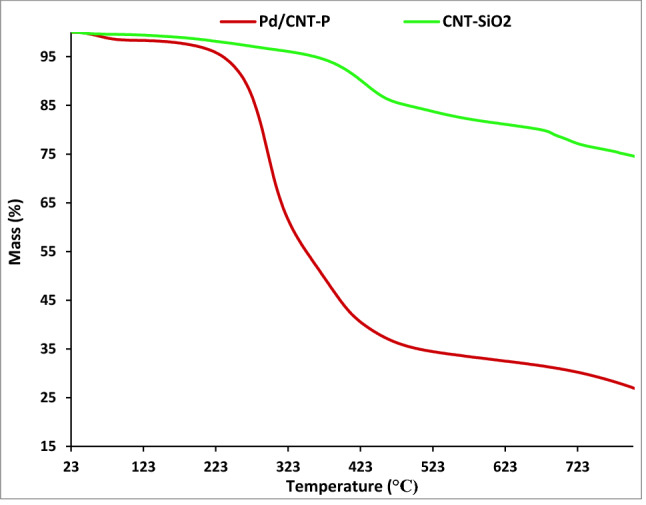
TG curves of CNT-SiO_2_ and Pd/CNT-P.

Thermogravimetic analysis of CNT-SiO_2_ and Pd/CNT-P samples has been conducted to compare their thermal stability and confirm conjugation of P. As depicted in Fig. 6, CNT-SiO_2_ showed high thermal stability. In fact, TG curve of CNT-SiO_2_ showed two weight loss stages related to the loss of water (150 °C) and degradation of CNT (above 550 °C). TG curve of Pd/CNT-P is distinguished from TG curve of CNT-SiO_2_. In this curve an additional weigh loss stage (44.3 wt.%) at 290 °C can be detected. This weigh loss is assigned to the degradation of P component.

**Figure 7:**
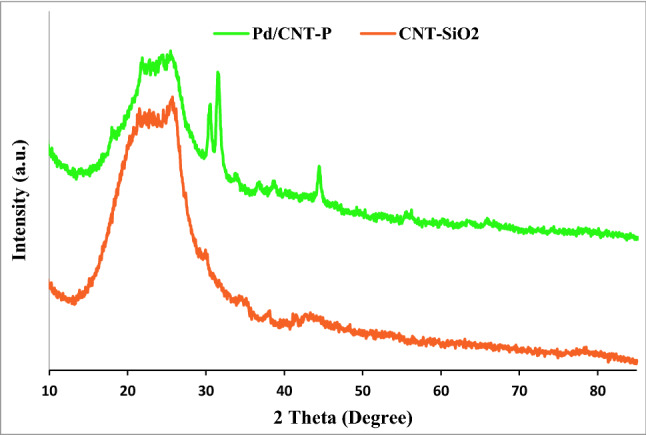
XRD patterns of Pd/CNT-P and CNT-SiO_2_.

Using XRD analysis, the structure of Pd/CNT-P was investigated. In this regard, the XRD pattern of Pd/CNT-P was recorded and compared with that of CNT-SiO_2_ (Fig. 7). The broad peak in the XRD pattern of CNT-SiO_2_ can be assigned to the amorphous silica coat and approve successful coverage of CNT with SiO_2_. Other small peaks can be attributed to CNT. In the XRD pattern of Pd/CNT-P, the CNT-SiO_2_ characteristic peaks can be detected. Noteworthy, the characteristic peak of the polymeric moiety (2θ = 15–25°) overlapped with the characteristic peaks of CNT-SiO_2_^19^. It is worth mentioning that the characteristic peaks of Pd nanoparticles were not discerned in the XRD pattern of Pd/CNT-P. This is assigned to the low content and high dispersion of Pd nanoparticles^46^.

### Activity of the catalyst

Hydrogenation of nitro compounds is an important chemical reaction. This reaction not only is used for the synthesis of anilines, but also is applied for the synthesis of key chemical intermediates that can potentially be utilized for the synthesis of complicated organic compounds and drugs. Considering the importance of this reaction, we examined the catalytic activity of Pd/CNT-P for the hydrogenation of nitro compounds. To initiate the experiments, hydrogenation of nitrobenzene was selected as a model hydrogenation reaction. In the first step, the optimum reaction condition was found by studying the effects of reaction solvent, temperature and Pd/CNT-P loading on the yield of aniline.

First, the effect of the reaction solvent was appraised. In this regard, hydrogenation of nitrobenzene at room temperature and 5 mg Pd/CNT-P was performed in H_2_O, EtOH, H_2_O:EtOH (1:1), CH_3_CN and THF. As tabulated in Table 1, the reaction in water led to slightly lower yield of aniline. In the other four solvents, similar yields of aniline were furnished, indicating that the nature of solvent did not have a significant effect on the yield of the reaction. Considering these results and environmental concerns, H_2_O:EtOH (1:1) was selected as the reaction solvent.

**Table 1:** Optimization of the reaction condition for the model reaction.

Entry	Pd/CNT-P (mg)	Solvent	Temp. (°C)	Yield (%)
1	5	H_2_O	25	60 ± 2
2	5	EtOH	25	70 ± 2
3	5	CH_3_CN	25	70 ± 1
4	5	THF	25	70 ± 3
5	5	H_2_O:EtOH (1:1)	25	70 ± 3
6	10	H_2_O:EtOH (1:1)	25	80 ± 2
7	15	H_2_O:EtOH (1:1)	25	80 ± 2
8	10	H_2_O:EtOH (1:1)	30	85 ± 3
9	10	H_2_O:EtOH (1:1)	40	98 ± 3

In the next step, the effect of Pd/CNT-P loading was examined by repeating the model reaction in the presence of 5, 10 and 15 mg of Pd/CNT-P loading. As summarized in Table 1, by increasing the loading of the catalyst to 10 mg, the reaction yield increased from 70 to 80%. However, more increment of Pd/CNT-P loading did not improve the reaction yield. Therefore, 10 mg Pd/CNT-P was selected as the best loading.

**Figure 8:**
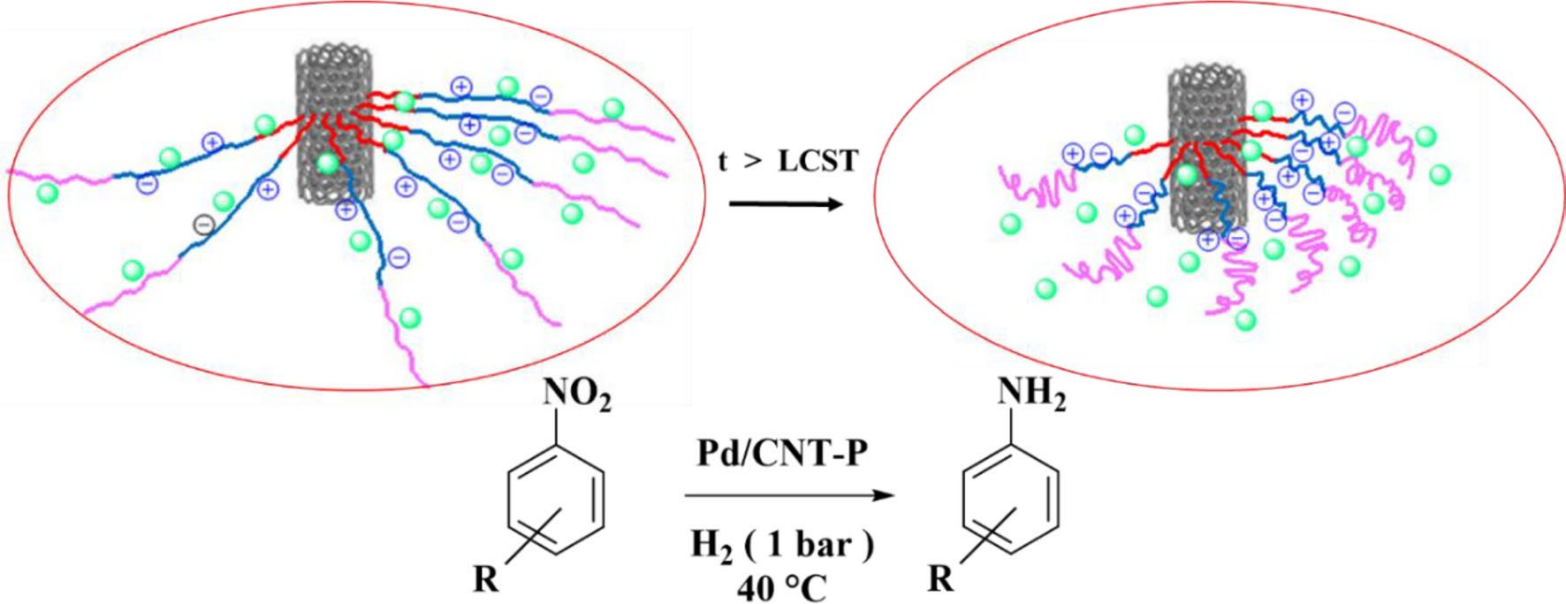
Schematic presentation of structural change of the thermo-responsive catalyst at temperature above LCST for the hydrogenation of nitro compounds.

Finally, the effect of the reaction temperature was investigated. To this purpose, the model reaction was conducted at 25, 30 and 40 °C. According to the results, elevating the reaction temperature was beneficiary for the reaction yield and the increase of the reaction temperature above LCST resulted in higher yield of aniline. This observation is in good accordance to the previous reports^10,47^. In fact, at temperature above the LCST (~ 37 °C), P component in the backbone of Pd/CNT-P collapses to furnish a hydrophobic periphery that is favorable for the mass-transfer of hydrophobic reactants, Fig. 8.

Having the optimum reaction condition in hand, other nitro compounds have been examined as substrates to confirm the generality of this protocol. As listed in Table 2, various nitro compounds can tolerate hydrogenation reaction under Pd/CNT-P catalysis to give the corresponding products in high yields. According to the results, the electronic feature of the substrates did not significantly affect the yield of the expected products and the substrates with both electron-donating and electron-withdrawing groups can furnish the products in high yields. However, steric effects can influence the reaction yield and huge and hydrophobic substrates led to lower yields. It is worth mentioning that Pd/CNT-P could catalyze the reaction of nitro compounds with carbonyl groups (4-nitroacetophenone and 4-nitrobenzaldehyde) selectively to give 4-aminoacetophenone and 4-aminobenzaldehyde as sole products. This observation approved high selectivity of Pd/CNT-P towards reduction of nitro functionality.

**Table 2:**
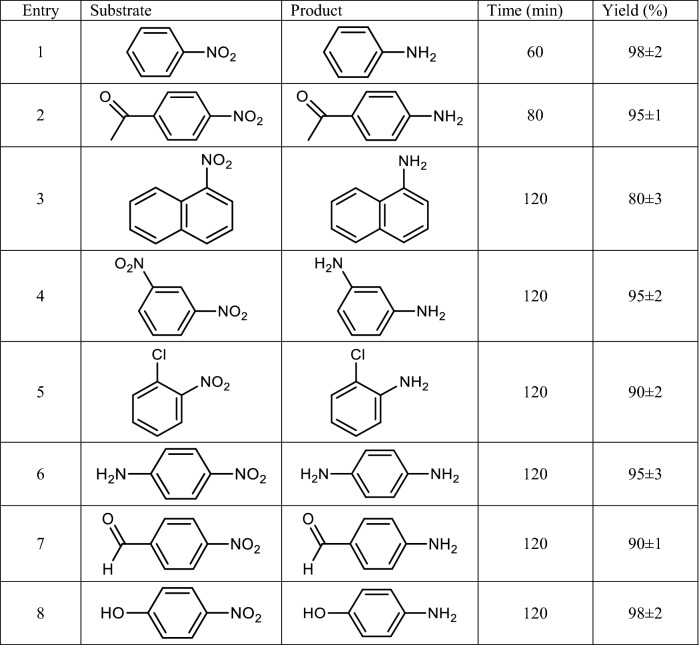
Hydrogenation of nitro compounds under Pd/CNT-P catalysis^a^.

^a^1 mmol nitro compound, H_2_O/EtOH (1/1), 40 °C under H_2_ gas (1 bar).

### Control catalysts

In this project, we aimed to design a thermos-responsive catalyst, which could promote hydrogenation of nitro-compounds in aqueous media efficiently. To this purpose, we conjugated P that is an IL-containing thermoresponsive polymer with CNT covalently to benefit from the characteristics of the two components and the possible synergism between them. To elucidate whether the activity of the as-prepared composite is superior to each components, several control catalysts, including, Pd/PNIPAM Pd/P, Pd/CNT-V-PNIPAM, Pd/CNT-V, were prepared (Figure S1) and their activity for the model reaction was compared with that of Pd/CNT-P, Table 3. First, Pd/CNT-V was synthesized through the exact procedure used for the palladation of CNT-P, except, CNT-V was applied as a support and its activity was evaluated for the model reaction under the optimum reaction condition. As shown in Table 3, Pd/CNT-V showed moderate activity and led to the formation of aniline in 45% yield. Moreover, this sample was not thermos-responsive and its recovery from the reaction media was tedious. This observation confirmed the necessity of the presence of P for achieving high catalytic performance.

**Table 3:** Comparison of the catalytic activity of Pd/CNT-P and control catalysts for the nitrobenzene hydrogenation.

Entry	Catalyst	Yield (%)^a^	Pd loading (wt.%)
1	Pd/PNIPAM	30	0.81
2	Pd/P	40	0.89
3	Pd/CNT-V-PNIPAM^b^	80	0.91
4	Pd/CNT-V	45	0.89
5	Pd/CNT-P	98	1

^a^Reaction condition: nitrobenzene (1 mmol), catalyst (10 mg), H_2_O:EtOH (1:1), H_2_ (1 bar) at 40 °C in 2 h.

^b^Pd was loaded on the CNT-V, which was polymerized with NIPAM.

Next, Pd/CNT-V-PNIPAM was prepared through polymerization of CNT-V and NIPAM, followed by palladation. The study of the catalytic activity of this sample that did not contain IL in its structure approved that its catalytic activity was lower than that of the catalyst. This result confirmed the importance of IL in the structure of the catalyst for achieving high activity.

Two other catalysts, Pd/PNIPAM and Pd/P were also prepared through palladation of PNIPAM and P. The catalytic studies showed that these two control samples that did not contain CNT exhibited moderate activity and led to the formation of aniline in 30 and 40% respectively. This observation affirmed the role of CNT in the catalysis. In conclusion, the higher activity of Pd/CNT-P compared to the studied control catalysts indicated the positive role of hybridization of P and CNT and the presence of IL in the catalytic activity. To appraise the origin of different catalytic activity of the control catalysts, they have been characterized via ICP. As shown in Table 3, the loadings of Pd in the control catalysts are different. In fact, it can be observed that the nature of the support can affect the capability of Pd loading. As this feature is an important factor on the catalytic activity of the catalyst, the control samples exhibited different activity. As tabulated, Pd/CNT-P possessed the highest Pd loading and showed the best catalytic activity. In Pd/CNT-V-PNIPAM, in which both CNT and polymeric moiety are present, the loading of Pd is also high and slightly lower than that of Pd/CNT-P. Hence, high catalytic activity was observed. In the case of other control catalysts, the Pd loadings were lower and moderate activity was detected.

### Recyclability

**Figure 9:**
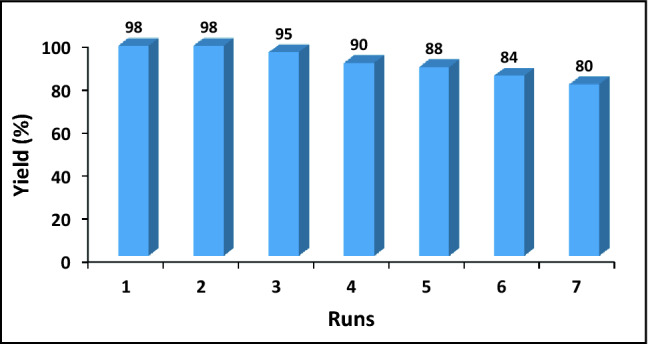
Recyclability of the catalyst for the model reaction under the optimum reaction condition.

As facile recovery and efficient recyclability are important factors for heterogeneous catalysts, recyclability of Pd/CNT-P for the model reaction was appraised. The recycling test was simply conducted by separation of Pd/CNT-P from the first run of the model reaction and its recovery through washing with toluene and drying at 50 °C in oven. The recovered Pd/CNT-P was then used as a catalyst for catalyzing the second run of the model reaction under similar condition. Upon completion of the reaction, the recovery-reuse cycle was repeated for seven consecutive runs. As shown in Fig. 9, Pd/CNT-P can be efficiently recycled and only slight loss of the activity was detected after each recycling.

**Figure 10:**
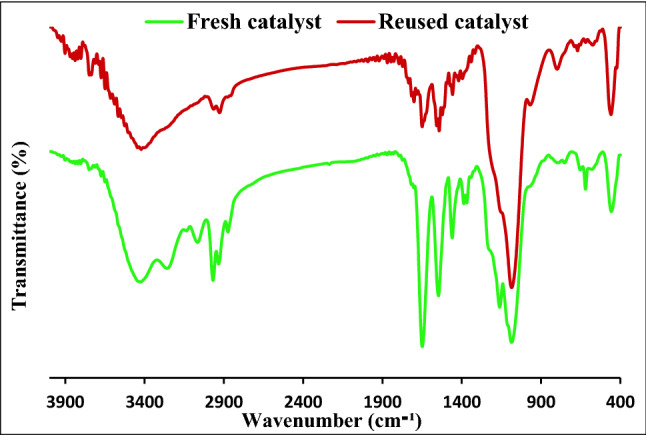
Comparison of the FTIR spectra of fresh and recycled catalyst after seven runs.

To study the stability of Pd/CNT-P structure upon recycling, the reused catalyst (obtained after seventh run) was characterized via FTIR spectroscopy. As shown in Fig. 10, the FTIR spectrum of the reused Pd/CNT-P exhibited all of the absorbance bands of fresh Pd/CNT-P. This observation affirms the stability of Pd/CNT-P upon recycling.

The measurement of leaching of Pd was performed via ICP analysis. To this purpose, the recovered catalyst after seventh run of the reaction was analyzed by ICP. It was found that Pd leaching was 1.6 wt.% of initial loading. According to these results and considering the fact that Pd species is the main component for promoting hydrogenation reaction, loss of the catalytic activity of the catalyst can be attributed to the Pd leaching.

**Figure 11:**
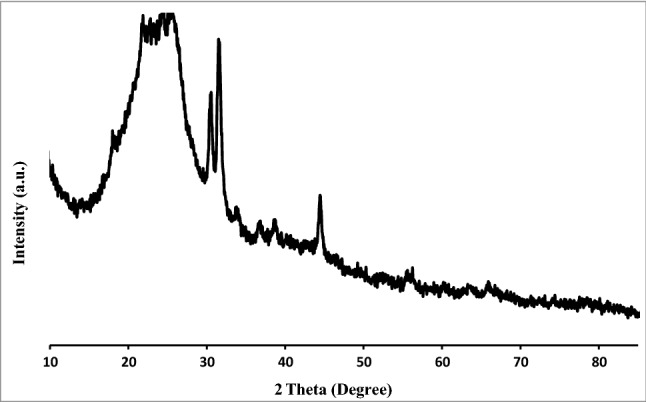
XRD analysis of the recycled catalyst after seven runs.

To appraise the stability of Pd/CNT-P in the course of recyclability, the recovered Pd/CNT-P after seventh run of the hydrogenation reaction was analyzed via XRD. As presented in Fig. 11, the recorded XRD pattern of the recovered Pd/CNT-P is identical to that of fresh one, confirming the stability of the catalyst under recovering-reusing cycles.

### Hot filtration test

There are two possibilities in heterogeneous catalysis. In true heterogeneous catalysis, it is expected that the catalytic species remains stabilized on the support, while in the second possibility, the catalytic species can leach to the reaction media and then stabilized on the support. The well-known test for determining the nature of the catalysis is hot filtration, in which the catalyst is removed from the reaction after a short period and then the reaction is pursued in the absence of the catalyst. In the case of true heterogeneous catalysis, in which no leaching of the catalytic species occurs, the reaction does not proceed after removal of the catalyst, while in the other pass way, the reaction proceeds in the absence of the catalyst due to the leached catalytic species. Hot filtration test approved that Pd/CNT-P was a true heterogeneous catalyst. In more detail, hydrogenation of nitrobenzene did not proceed after removing of Pd/CNT-P from the reaction vessel.

### Comparative study

The activity of Pd/CNT-P for the hydrogenation of nitrobenzene was compared with some of the Pd-based catalysts, reported in the literature, Table 4. As illustrated, Pd nanoparticles have been suppoerted on various supporting materials. Moreover, different reaction conditions have been reported using the tabulated catalysts. Although in some catalysts, availble and low-cost supports, such as Al_2_O_3_ and sucrose nanoparticles has been reported, high hydrogen pressure or toxic solvents were used for achieving high yield of aniline. In some reports, halloysite nanoclay has been utilized for the immobilization of Pd nanoparticles. Although halloysite is a natural clay, it is relatively costly. On the other hand, mostly, halloysite is modified chemically through multi-step procedures, using toxic reagents and solvents prior to Pd stabilization. This issue negatively affects the cost of the final catayst. Metal organic frameworks have also been employed as a support. In this case, the required hydrogen pressure was high that is not favorable from environmental point of view. On the other hand, synthesis of metal organic frameworks is a delicate process, using some costly linkers.

**Table 4:** The comparison of the activity of Pd@Per-P for the model hydrogenation reaction.

Entry	Catalyst	Solvent	H_2_ Pressure	Time (min)	Temp. (°C)	Yield (%)	Refs.
1	PdNP(0.5%)/Al_2_O_3_ (0.3 g)	THF	1 atm	180	r.t	100	^48^
2	Pd@Hal-Hydrogel + cyclodextrin (2 wt.%)	H_2_O	1 bar	120	50	95	^49^
3	Pd@Hal-TCT-Met^a^	H_2_O	1 bar	75	65	100	^50^
4	APSNP ^b^ (1 mol%)	EtOH	40 atm	120	r.t	100	^51^
5	Pd@CS-CD-MGQDs^c^ (0.5 mol%)	H_2_O	1 atm	60	50	97	^52^
6	Pd/PPh_3_@FDU‐12 (8.33 × 10^–4^ mmol Pd)	EtOH	10 bar	60	40	99	^53^
7	Pd@Hal-biochar^d^ (0.03 mol%)	H_2_O	1 bar	60	r.t	75	^54^
8	Pd@Hal/di‐urea^e^ (1.5 wt.%)	H_2_O	1 atm	60	50	100	^55^
9	Pd@Per-P(0.03 g)	H_2_O:EtOH (1:1)	1 atm	90	45	98	^56^
10	Pd@Hal-CCD^f^ (1 wt.%)	H_2_O	1 atm	90	r.t	100	^57^
11	Pd/CNT-P	H_2_O:EtOH (1:1)	1 bar	120	40	98	This work

^a^Pd immobilization on the multi-amine functionalized halloysite.

^b^Activated palladium sucrose nanoparticles.

^c^Pd on hybrid of magnetic graphene dots and cyclodextrin decorated chitosan.

^d^Hybrid of halloysite and char.

^e^Halloysite clay decorated with ligand.

^f^Halloysite decorated with cyclodextrin derived carbon sphere.

### Reaction mechanism

**Figure 12:**
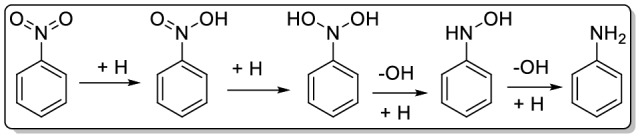
The proposed mechanism for the hydrogenation of nitrobenzene.

According to the previous reports, the possible mechanism for the hydrogenation of nitroarenes can be depicted as Fig. 12^58^. As illustrated, hydrogenation reaction proceeds via double H-induced dissociation of N–O bond, in which the Pd species on the CNT-P can play role in activation of the substrate.

## Experimental section

### Materials and instruments

COOH-functionalized multi-wall carbon nanotubes (MWCNTs) (purity > 95%, content of COOH = 3.86 wt.%, inner diameter: 2–5 nm and outer diameter > 7 nm) were obtained from US Research Nanomaterials. Other reagents and solvents applied for the synthesis of the catalyst and conducting hydrogenation reaction of nitro-compounds are as follow: (3-trimethoxysilyl) propyl methacrylate (TMSPMA), cetyltrimethyl ammonium bromide (CTAB), tetraethyl orthosilicate (TEOS), 1-vinylimidazole, NIPAM, azobisisobutyronitrile (AIBN), triethylamine (Et_3_N), palladium(П) acetate Pd(OAc)_2_, sodium borohydride (NaBH_4_), *N,N*-dimethylformamide (DMF), nitro-compounds, 1-bromo butane, sodium hydroxide, toluene, ethanol (EtOH) and methanol (MeOH), all was purchased from Sigma-Aldrich ( Germany, Taufkitchen) and used without further purification.

The formation of Pd/CNT-P was confirmed by various characterization techniques, including: Fourier transform Infrared spectroscopy (FT-IR), X-ray diffraction (XRD), thermogravimetric analysis (TGA), transmission electron microscopy (TEM), inductively coupled plasma mass spectroscopy (ICP-MS), energy-dispersive X-ray spectroscopy (EDS) and elemental mapping analysis.

Recording of FTIR spectra was carried out by using BRUKER TENSOR 35 spectrophotometer 65 with scan time of 1 s and spectral resolution of 2 cm^−1^ by using KBr pellets. The XRD pattern of Pd/CNT-P was recorded using a Rigaku Ultima IV with Cu Kα radiation from a sealed tube. Thermal stability of Pd/CNT-P was investigated by applying METTLER TOLEDO thermogravimetric analysis apparatus. To record the catalyst thermogram, inert atmosphere and heating rate of 10 °C min^−1^ were applied. TEM images of Pd/CNT-P were recorded on a Phillips EM 208S microscope at 100 kV. Pd loading of Pd/CNT-P was estimated using ICP analyzer Varian, Vista-pro instrument. EDX and elemental mapping analyses were carried out by Tescan Mira II.

## Preparation of Pd/CNT-P

### Surface modification of carbon nanotubes: synthesis of CNT-SiO_2_

Surface modification of CNT with silica was performed according to the previous report^59^. Typically, to a solution of CTAB (1.7 g) in distilled water (40 ml), CNT (0.2 g) was added and the resulting suspension was homogenized under ultrasonic irradiation (160 W for 30 min). Afterwards, NaOH (0.08 g) was added and the resulting mixture was stirred for 30 min at 60 °C. In the next step, a solution of TEOS (10 mL) in EtOH was introduced to the aforementioned suspension under stirring condition at 60 °C. After 24 h, the product, CNT-SiO_2_, was separated via centrifugation, washed repeatedly with warm EtOH and dried in oven overnight.

### Vinyl-functionalization of CNT-SiO_2_: synthesis of CNT-V

In order to vinyl-functionalize CNT-SiO_2_, the as-prepared CNT-SiO_2_ (0.5 g) was dispersed in dry toluene (20 mL) by using ultrasonic irradiation (160 W) for 20 min. Then TMSPMA (0.5 g) was added to CNT-SiO_2_ suspension and the resulting mixture was refluxed at 110 °C for 24 h under Ar atmosphere. Upon completion of the reaction, the precipitate, CNT-V, was collected via centrifuge, washed with toluene and dried in oven at 80 °C overnight.

### Synthesis of VBIB

VBIB was prepared according to the previous report^60^. Briefly, the mixture of 1-bromobutane (4 g) and 1-vinylimidazole (2 g) was stirred at 70 °C under solvent-free condition for 16 h under inert atmosphere. Then, the reaction mixture was allowed to cool to room temperature and the product was collected, washed several times with ethyl acetate and dried in a vacuum oven overnight.

### Polymerization of NIPAM, VBIB and CNT-V: synthesis of CNT-P

CNT-V (0.5 g) was added to a solution of NIPAM (0.5 g) in DMF (20 mL) under stirring condition under Ar atmosphere at 70 °C. After 20 min, a solution of AIBN (0.15 g) in DMF (10 mL) was added to the aforesaid mixture in a dropwise manner and stirring was maintained for 40 min. Subsequently, a solution of VBIB (0.5 g) in DMF (10 mL) was added and the resulting mixture was stirred for 24 h at 70 °C under Ar atmosphere. After completion of the reaction, the solvent was evaporated and the solid was collected, rinsed with DMF and dried in oven at 70 °C overnight.

### Immobilization of Pd nanoparticles on CNT-P: Synthesis of Pd/CNT-P

In order to prepare Pd/CNT-P, CNT-P (1.5 g) was stirred in toluene (20 mL) in a 2-neck round flask under Ar atmosphere at room temperature for 30 min. Subsequently, a solution of Pd(OAc)_2_ (0.02 g) in toluene (15 mL) was gently introduced to the aforementioned suspension. After 1 h, a solution of NaBH_4_ (0.2 N) in MeOH (20 mL) was slowly added and the obtained mixture was stirred for 2 h. Finally, the as-prepared Pd/CNT-P was collected via centrifugation, washed with toluene and dried in oven at 50 °C overnight, Fig. 1. According to the ICP analysis, Pd loading in Pd/CNT-P was 1 wt.%.

### Typical procedure for the hydrogenation of nitro-compounds

Hydrogenation of nitro-compounds was conducted under mild reaction condition. Typically, a mixture of nitro aromatic compound (1 mmol) and Pd/CNT-P (0.01 g) in H_2_O/EtOH (1/1) was heated to 40 °C under H_2_ gas (1 bar). The progress of the hydrogenation reaction was traced by TLC. Upon completion of the process, Pd/CNT-P was separated and recovered by washing with toluene (30 mL) and drying at 50 °C in an oven overnight. The recovered catalyst was reused for the next run of the reaction. The hydrogenized product was obtained after evaporation of the solvent. The reaction yield was measured via GC analysis.

## Conclusion

A novel thermo-responsive catalyst, Pd/CNT-P, was designed and synthesized through vinyl functionalization of CNT-SiO_2_, followed by polymerization with NIPAM and VBIB and palladation. The catalytic performance of the resulting catalyst was examined for the hydrogenation of nitro-compounds in aqueous media under mild reaction condition. The results affirmed high catalytic activity of the catalyst, broad substrate scope and high selectivity of the catalyst towards nitro group. Moreover, the catalyst was recyclable up to seven reaction runs with slight Pd leaching. Hot filtration test also indicated the true heterogeneous nature of the catalysis. The comparison of the catalytic activity of Pd/CNT-P with several control catalysts confirmed the importance of conjugation of P and CNT for achieving high catalytic activity.

## Supplementary Information

Supplementary Information.
