# Supplementary material for: Pd on thermo-responsive composite of silica-coated carbon nanotube and 1-vinyl-3-butylimidazolium-based ionic liquid copolymers as an efficient catalyst for hydrogenation of nitro compounds
Source: Sci Rep. 2022 Mar 10;12:3972. doi: 10.1038/s41598-022-07708-0 (PMC8913645; doi:10.1038/s41598-022-07708-0)
Supplement: Supplementary file 1 — Supplementary Information. [file 41598_2022_7708_MOESM1_ESM.docx]

**Supporting information**

**Pd on thermo-responsive composite of silica-coated carbon nanotube and 1-vinyl-3-butylimidazolium-based ionic liquid copolymers as an efficient catalyst for hydrogenation of nitro compounds**

Samahe Sadjadi^1*^, Neda Abedian-Dehaghani^2^, Majid M. Heravi^*2^

**
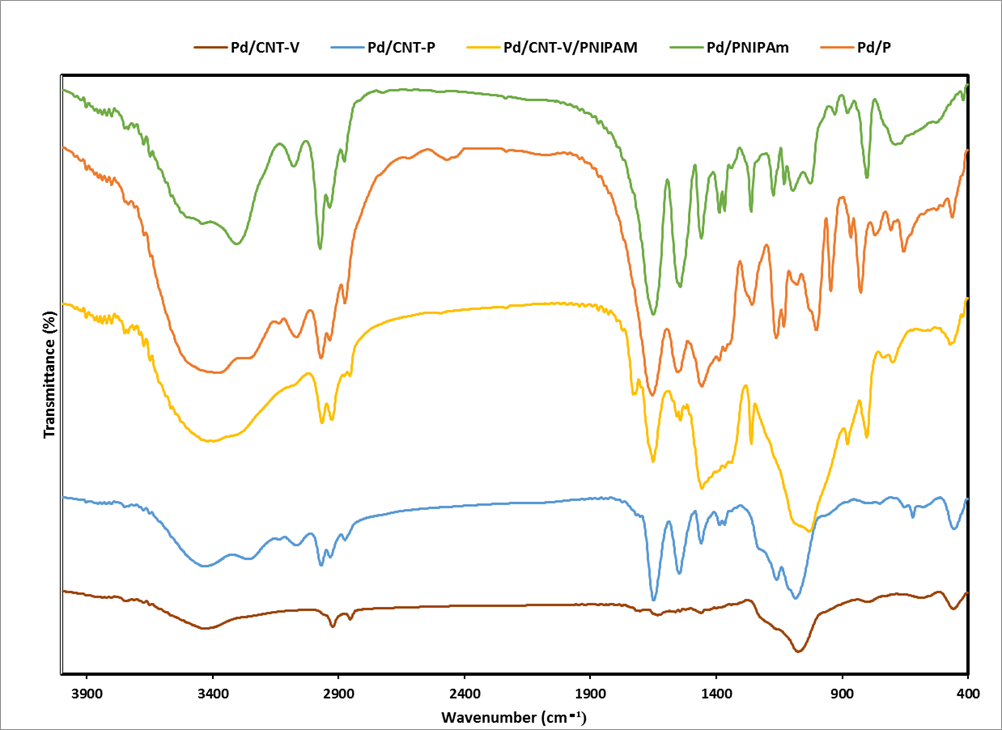
**

**Figure S1.** FTIR spectra of the control catalysts.
